# Isoprenaline and salbutamol inhibit pyroptosis and promote mitochondrial biogenesis in arthritic chondrocytes by downregulating β-arrestin and GRK2

**DOI:** 10.3389/fphar.2022.996321

**Published:** 2022-09-14

**Authors:** Iqra Ajmal, Muhammad Asad Farooq, Syed Qamar Abbas, Jaffer Shah, Muhammad Majid, Wenzheng Jiang

**Affiliations:** ^1^ Shanghai Key Laboratory of Regulatory Biology, School of Life Sciences, East China Normal University, Shanghai, China; ^2^ Department of Pharmacy, Sarhad University of Science and Technology, Peshawar, Pakistan; ^3^ Department of Health, New York, NY, United States; ^4^ Faculty of Pharmacy, Capital University of Science and Technology Islamabad, Islamabad, Pakistan

**Keywords:** rheumatoid arthritis, chondrocytes, β2-adrenergic receptor, isoprenaline, salbutamol

## Abstract

Rheumatoid arthritis and osteoarthritis overlap many molecular mechanisms of cartilage destruction. Wear and tear in cartilage is chondrocyte-mediated, where chondrocytes act both as effector and target cells. In current study, role of β2-AR was studied in chondrocytes both *in vitro* and *in vivo*. High grade inflammation *in vitro* and *in vivo* disease models led to decline in anti-inflammatory β2-AR signaling and use of β2-AR agonist attenuated arthritis symptoms. Detailed analysis in chondrocytes revealed that Isoprenaline (ISO) and Salbutamol (SBT) increased cell viability and relative Bcl-2 expression, meanwhile, decreased proteins levels of TNF-α, IL-6 and IL-8 in arthritic chondrocytes when compared with control, respectively. SBT preserved physiological concentration of antioxidant enzymes (CAT, POD, SOD and GSH) in cartilage homogenates and ISO inhibited IL-1β-mediated genotoxicity in arthritic chondrocytes. Moreover, β2-AR agonist increased mitochondrial biogenesis and proteoglycan biosynthesis by upregulating the gene expression of *PGC1-α*, *NRF2* and *COL2A1*, *Acan,* respectively. ISO and SBT inhibited extracellular matrix (ECM) degradation by downregulating the gene expression of *MMP1*, *MMP3, MMP9* and *ADAMTS5 in vitro* and *in vivo* study. In mechanism, β2-AR agonists decreased β-arrestin and GRK2 pathway, and as a result mice receiving SBT did not exhibit severe disease. Hence our data suggest β2-AR agonist administered at disease onset can inhibit receptor internalization by downregulating the expression of β-arrestin and GRK2 in chondrocytes.

## Introduction

Rheumatoid arthritis (RA) affects roughly 5 out of every 1,000 people and it can cause substantial inflammation and disabilities of joints. Over the last 2 decades, great strides have been made in terms of pathogenesis and treatment strategies of the disease (Reginster, 2002; Aletaha and Smolen, 2018). Inflammatory mediators released in the synovial fluid and synovium as a result of the RA are directly and indirectly influencing the homeostasis of the cartilage. Chondrocytes are cartilage-specific cells that act both as target and effector cells during cartilage degeneration process (Robbins et al., 2000; Ota et al., 2020). Despite being one of the damaged cells in RA, chondrocytes also go through molecular changes that cause them to actively contribute to inflammation and matrix degradation in the RA affected joints (Tseng et al., 2020).

Dysregulation of neuro-immune crosstalk in autoimmune diseases is the hallmark of several autoimmune disorders. Dysfunctional immune system in autoimmune disorders leads to loss of anti-inflammatory β2- adrenergic receptors (β2-ARs) in RA, which can further worsen the disease state (Levine et al., 1988). β2-ARs are expressed on variety of immune cells and chondrocytes. Typically, β2-AR signal through cyclic AMP (cAMP) pathway that is anti-inflammatory. In extreme inflammatory conditions, desensitization of β2-AR takes place by protein kinase A (PKA) phosphorylation, and receptor signaling starts in a β-arrestins and GRKs-dependent manner in immune cells as well as in immune organs (Wahle et al., 1999; Lorton and Bellinger, 2015; Wu et al., 2018).

In RA rat model, weaker β2-AR signaling was found in bone marrow derived dendritic cells. Administration of salbutamol in arthritic rats attenuated arthritis symptoms with lesser disease scores signifying that control of disease was due to agonist mediated increased antigen uptake by dendritic cells (DCs). β2-AR agonists in RA are reported to downregulated proinflammatory cytokine production and increase anti-inflammatory cytokines, as well as decreasing T cell infiltration to the synovium (Lubahn et al., 2014; Bellinger et al., 2021). So role of β2-AR in arthritis is well established.

Recently, there has been reports about the involvement and criticality of chondrocytes in arthritis as they are demonstrated to release inflammatory mediators in arthritic synovium (Otero and Goldring, 2007). β2-ARs are reported to be expressed in chondrocytes as well. There are only fewer studies regarding the role that β2-ARs play in these important set of cells i.e. chondrocytes. Previously, a study by Lick Puj Lai, and Jane Mitchell demonstrated isoproterenol as an important regulator and maintainer of cartilage hemostasis. The authors concluded that adrenergic signaling is vital for cartilage development at embryonic stage (Lai and Mitchell, 2008). Given that β2-AR are expressed in chondrocytes, and have their role in cartilage development, we tried to explore the detailed role of β2-AR agonists’ salbutamol and Isoprenaline in arthritic chondrocytes. In present study, we sought to demonstrate that β2-AR signaling is dysregulated in chondrocytes both *in vitro* and *in vivo* just like in immune cells, and use of β2-AR agonist in the early phase of arthritis can prevent high grade inflammation and hence receptor desensitization in chondrocytes can be prevented. This was proven to be beneficial for overall chondrocyte metabolic and functional fitness in both human chondrocyte cell lines as well as in murine chondrocytes.

## Material and methods

### Cell line and cell culture

Human chondrocyte cell line CHON-001 was purchased directly from American type culture collection (ATCC, CRL-2846). Briefly, CHON-001 cells were thawed and cultured immediately in a humid incubator (5% CO_2_, 37°C) after receipt according to the instructions. The cells were cultured and maintained in DMEM media (Hyclone, Logan, UT, United States) containing 10% FBS and 1% penicillin/streptomycin. When cells reached 80% confluence, they were subcultured at a 1:4 ratio. Cells were collected from the petri plate for subculturing by using 2–3 ml 0.05% Trypsin-0.53 mm EDTA (Cat # 25200056) (Hyclone, Logan, UT, United States). All the experiments in this study is performed after three passages. Cells were cryopreserved with Fetal Bovine Serum (FBS) (Hyclone, Logan, UT, United States) supplemented with 10% DMSO in liquid nitrogen.

*In vitro* experimental design consisted of 4 groups. For each group, 1 × 10^5^ chondrocyte cells were seeded in 48-well plate in duplicate. Group 1 was the control group without any treatment for the cells. Group 2 was treated with IL-1β (10 ng/ml) for 24 h. Group 3 was treated with β2-AR agonist Isoprenaline (ISO) (10^−5 ^mol/L) purchased from Sigma (St. Louis, MO, United States) for 24 h. Group 4 was treated with IL-1β for 6 h and then ISO was administered up to 24 h in total. Cells were collected to perform the further experiments.

### Animal model

In this study, Swiss albino mice (8–12 weeks old) were used. Mice were purchased from National Institute of Health (NIH, Islamabad, Pakistan). The mice employed in this study were all healthy, active, and exhibited no signs of physical impairment. They were housed in a pathogen-free environment at 22°C, with ample food and water provided with *ad libitum*. Experiment was performed following strict instruction and guidelines by Ethics committee Board Capital University of science and technology (Letter # CUST-PHM-018/2016).

Mice were divided into three groups (*n* = 8). Control groups included saline and mineral oil (MO). Study group received commercially available Complete Freund adjuvant (CFA) purchased from Thermo Fisher scientific (CAT # 77140) and was kept at 4°C. To make a perfect emulsion, equal amounts of immunogen and Freund adjuvant were blended according to the manufacturer’s instructions. CFA (50 µL) was injected slowly into the tail tissue, keeping a distance of 1.5 cm from the tail base and avoiding any surrounding arteries. Each paw was evaluated individually on a scale of 1-5 according to previously reported protocol and disease scores were recorded (Phull et al., 2017). Mice were euthanized at day 28 Post immunization after being given general anesthesia with (IP) 400 mg/kg body weight chloral hydrate (Sigma, St. Louis, MO, United States), chondrocytes were extracted and isolated.

In separate experiment, mice were divided into six groups (*n* = 20) and arthritis was induced in all of them except control group.

Group 1: Control group receiving vehicle.

Group 2: Arthritic mice.

Group 3: Arthritic mice receiving 0.75 mg/kg body weight of SBT.

Group 4: Arthritic mice receiving 1.5 mg/kg body weight of SBT.

Group 5: Arthritic mice receiving 3 mg/kg body weight of SBT.

Group 6: Arthritic mice receiving 0.5 mg/kg body weight of MTX.

All the treatment groups received respective agents from day 14 postimmunization. Commercial grade salbutamol (Ventolin GlaxoSmithKline) and Methotrexate (High noon laboratories Lahore Pakistan) were administered intragastrically every 3 days starting from day 15. Mice were monitored and disease scores were measured as described by our previous studies (Phull et al., 2017). Mice were euthanized at day 1 (*n* = 2), 7 (*n* = 2), 14 (*n* = 2), 21 (*n* = 2) and 28 (*n* = 12) and chondrocytes (*n* = 8) and cartilage homogenates (*n* = 4) were isolated according to protocol mentioned below.

### Isolation of murine chondrocytes

Isolation of murine chondrocytes involves two steps. 1) Isolation of articular cartilage; 2) isolation of chondrocytes from cartilage. Briefly, mice were located in inverted position with their heads down on a paper with their legs fixed with needle after general anesthesia. After removing skin and soft tissues from legs, target joints were dislocated and soft tissue were discarded. Articular cartilage was isolated and washed twice with PBS. Then cartilage chondrocytes were prepared by enzymatic digestion, and were cultured into DMEM supplemented with 10% FBS, 1% P/S. After 24 h, cells were collected for further experiments.

### Trypan blue cell exclusion method

CHON-001 cells and murine chondrocytes from all the experimental groups were stained with trypan blue to determine the numbers of dead and alive cells as described by Gosset and his colleagues in 2008 (Gosset et al., 2008). In a cuvette, 90 µL Trypan blue solution (0.4% wt/vol) and 10 µL cell suspension were prepared, and the hemocytometer was used to load the samples. Dead cells were identified by their blue tint, which absorbed the dye and radiated it. Hemocytometer count the number of dead, living, and total cells in the sample. The following formula was used to calculate cell viability and apoptosis rate, respectively.

$$\%\;viable\;cells=\frac{\#\;of\;viable\;cells}{\#\;of\;total\;cells}\times 100$$

Apoptosis rate=100−%viable cells

### Antioxidant assay

Mice (*n* = 4) from each group were euthanized and articular cartilage was isolated. Cartilage homogenates were made, centrifuged and serum was carefully collected by Pasteur pippete. CAT, POD, SOD, GSH were measured in the serum according to our previous protocol mentioned by Ul Hassan and Mahmood in their respective study (Ul Hassan et al., 2021; Mahmood et al., 2022). Briefly, rate of hydrolysis of H_2_O_2_ was measured spectrophotometrically at 240 nm for measuring serum catalase. Activity of superoxide dismutase in serum was measured by quercetin auto-oxidation inhibition method.

### Comet Assay

CHON-001 cells were treated with IL-1β, ISO or IL-1β+ISO for 24 h. Cells were centrifuged, media were removed and cells were resuspended in PBS. Very few cells were taken randomly and were fixed on 1% low melting point agarose gel slides and were subjected to lysis buffer for an hour. Afterwards agarose slides were kept in alkaline solution and subsequently were run across electrophoresis panel (Tang et al., 2018; Majid et al., 2022). Comet images were analyzed by CASPLAB software.

### Quantitative real time PCR analysis

Quantitative PCR was used to examine the gene expression level of various molecules such as *β2-AR*, *Bcl-2*, *GLUT-1*, *PGC-1α*, *NRF2*, *MMP1*, *MMP3*, *MMP9*, *ADAMTS5*, β-arrestin and *GRK2.* Chondrocytes were collected, washed, and resuspended in PBS from *in vitro* and *in vivo* experimental groups. The cells were suspended in 1 ml of Trizol reagent and were placed on ice, mRNAs were extracted according to the manufacturers’ instructions. The mRNAs were reverse transcripted and converted to cDNAs using the Invitrogen Superscript II Reverse Transcriptase Kit (Cat # 18064071). The purity and concentration of cDNA were assessed with Nanodrop. 500 ng cDNA was then used to perform the qPCR using SybrGreen based qPCR master mix (Cat #K0243) and their respective primers. Primers were designed from Primer bank by using the GenBank accession number. Primer sequence can be found in supplementary material (Supplementary Table S1). The qPCR process was set up as follows: 15 min of denaturation at 95°C, followed by 40 cycles of 95°C for 30 s, 30 s of particular primer annealing temperature, and 30 s of amplification at 72°C. For normalization, GAPDH, a housekeeping gene, was selected as a reference gene. Control group either from *in vitro* or *in vivo* were used to compare the fold change and set to 1. Relative expression was measured by 2^−ΔΔct^ method. In all experiments, samples were loaded in triplicate manner. Results were analyzed and graph was prepared by using Graph Pad Prism version 7.0.

### Cytokine quantification by ELISA

CHON-001 cells were cultured in complete DMEM medium. Supernatants were collected after 24 h and stored at −80°C. For analysis, 50 µL supernatants were used for IL-6 (Cat # EH2IL6), IL-8 (Cat # KHC0081) and TNF-α (Cat # BMS223INST) protein analysis according to manufacturer’s instruction. Murine chondrocytes were isolated from each experimental group and cultured for 24 h. Supernatants were collected for protein analysis of IL-6 (Cat # BMS603-2), IL-8 (Cat # EMCXCL15) and TNF-α (Cat # BMS607-3) according to manufacturer’s instructions. All the ELISA kits were purchased from Thermo fisher scientific.

### Statistical analysis

The data is presented as a mean ± standard deviation (SD). One-way ANOVA, or Two-way ANOVA, followed by the Bonferroni multiple comparison test, were used to determine statistical differences between groups. Statistical significance was defined as *p* < 0.05. GraphPad Prism version 7.0 for Windows was used for statistical analysis (GraphPad Software).

## Results

### β2-AR is downregulated in arthritic chondrocytes

**FIGURE 1:**
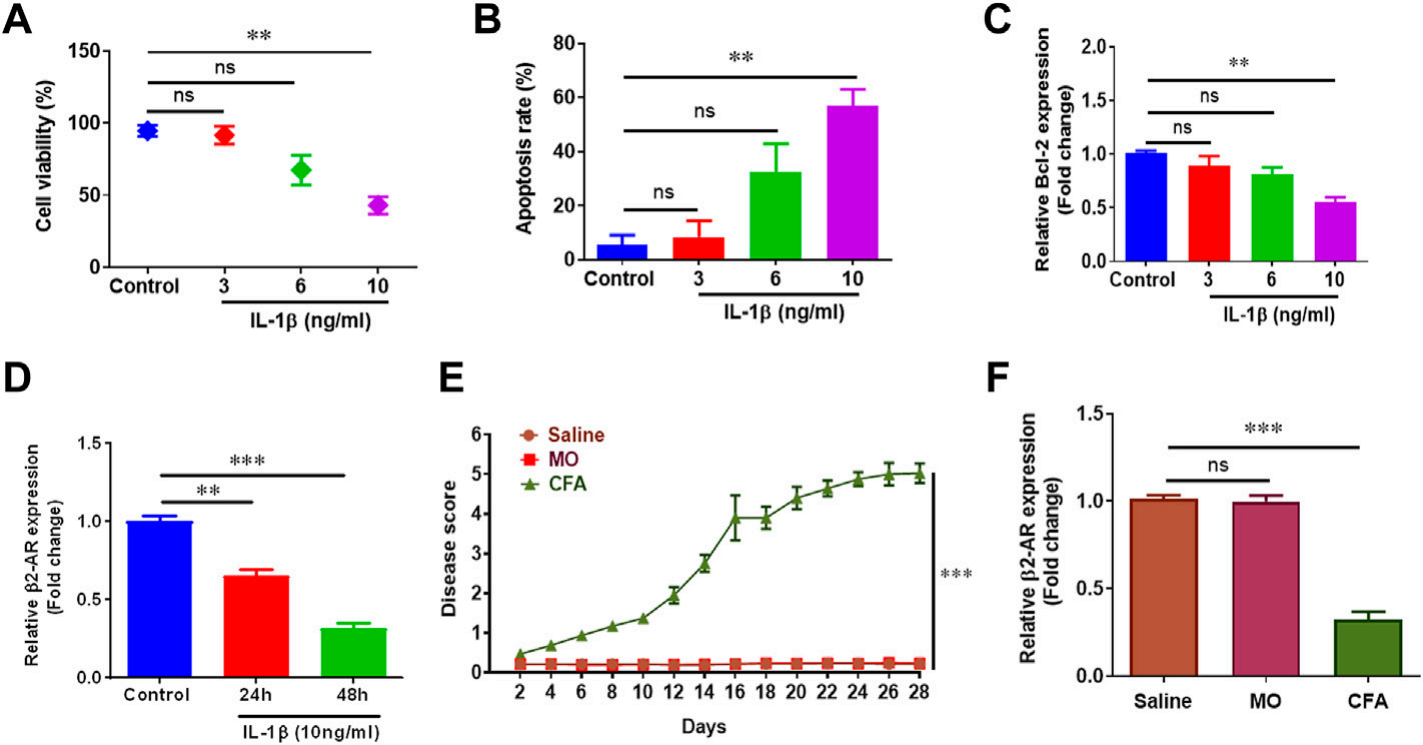
Inflammation reduces β2-AR expression. 1 × 10^5^ CHON-001 cells were seeded in 48 well plate and treated with 3 ng/ml, 6 ng/ml and 10 ng/ml IL-1β except control group for 24 h. Cell viability **(A)** and apoptosis rate **(B)** was determined by using trypan blue exclusion method. Total RNAs were extracted and qPCR was performed for measuring the gene expression of *Bcl-2* **(C)** and *β2-AR* **(D)**. **(E)** Swiss albino mice (*n* = 8) were injected intradermally with saline, MO and CFA. Mice were monitored every other day and disease score was calculated. **(F)** Mice were sacrificed at the end of the experiment at day 28, chondrocytes were isolated and gene expression level of *β2-AR* was measured by qPCR. GAPDH was used as a reference in this study. All the experiments were performed in duplicates and results were prepared by using Graph Pad Prism version 7.0. Significance was measured by using One Way ANOVA. **p < 0.01, ***p < 0.001, ns, not significant.

Chondrocytes constitute only 5–10% of the total cartilage volume but are extremely crucial in the pathogenesis of arthritis (Tseng et al., 2020). IL-1β has been known to play vital role in cartilage destruction in arthritis by mediating various pro-inflammatory mediators of inflammation, and is used to simulate arthritis *in vitro* (Ruscitti et al., 2018). Human chondrocyte cell line CHON-001 was treated with different doses of IL-1β to optimize *in vitro* disease model. IL-1β (10 ng/ml) significantly inhibited cell viability, promoted apoptosis and inhibited *Bcl-2* gene expression in CHON-001 cells and was used in following experiments (Figures 1A–C). Further, we found the expression of β2-AR receptors was significantly downregulated in CHON-001 cells treated with IL-1β (10 ng/ml) in a time-dependent manner. (Figure 1D). These findings were further confirmed in *in-vivo* disease model. Briefly, mice were divided into three groups (*n* = 4) and were administered saline, mineral oil (MO) and Complete Freund’s adjuvant (CFA), and disease scores were recorded (Figure 1E). Saline-treated group served as a control for handling and injection stress, while MO was used as a control for CFA vehicle. Here we found that β2-AR gene expression was also downregulated in chondrocytes of arthritic mice as shown in our qPCR data (Figure 1F).

### β2-AR agonist salbutamol attenuates adjuvant arthritis in mice

**FIGURE 2:**
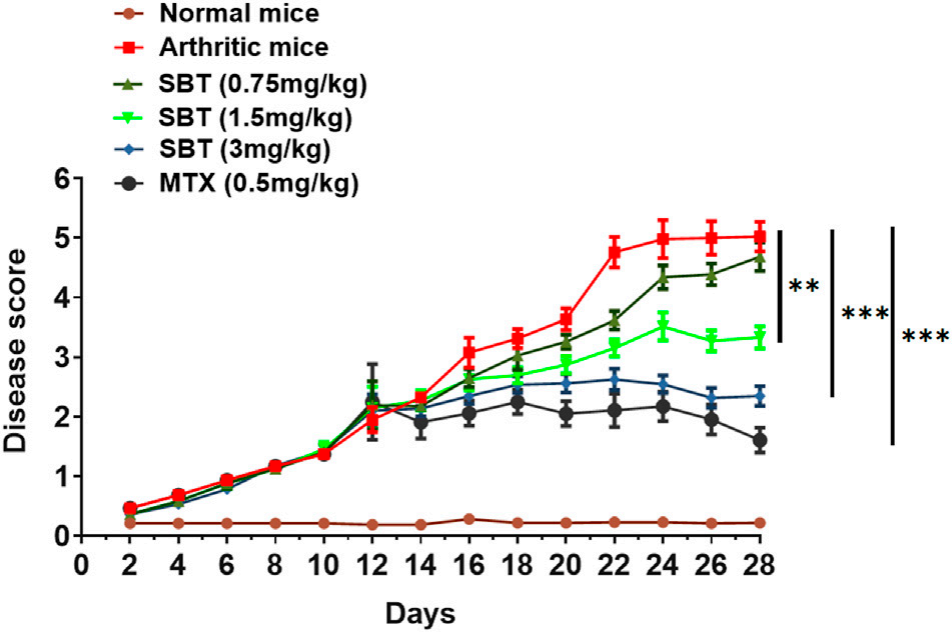
SBT decreases disease severity in mice. Disease score curve. CFA was administered to the study groups at day 0. After 15 days post immunization, the mice (*n* = 20) were administered with drugs either SBT (0.75 mg/kg, 1.5 mg/kg and 3 mg/kg) or MTX (0.5 mg/kg) and disease score was recorded. Disease score curve was prepared by using GraphPad Prism version 7.0. Results were analyzed using Two Way ANOVA with Bonferroni’s multiple comparison test. **p < 0.01, ***p < 0.001.

Pharmacological intervention was carried out to further decipher β2-AR role in CFA model. Salbutamol (SBT, 0.75 mg/kg) did not make a notable change in disease score but SBT (1.5 mg/kg and 3 mg/kg) attenuated arthritis symptoms and a remarkable decline in arthritic score was observed (Figure 2). All the treated groups either receiving SBT or MTX were administered drugs from day 14 post-immunization, the results demonstrated that administration of β2-AR agonist attenuated arthritis symptoms from day 15.

### ISO and SBT decrease apoptosis and increase survival of arthritic chondrocytes

**FIGURE 3:**
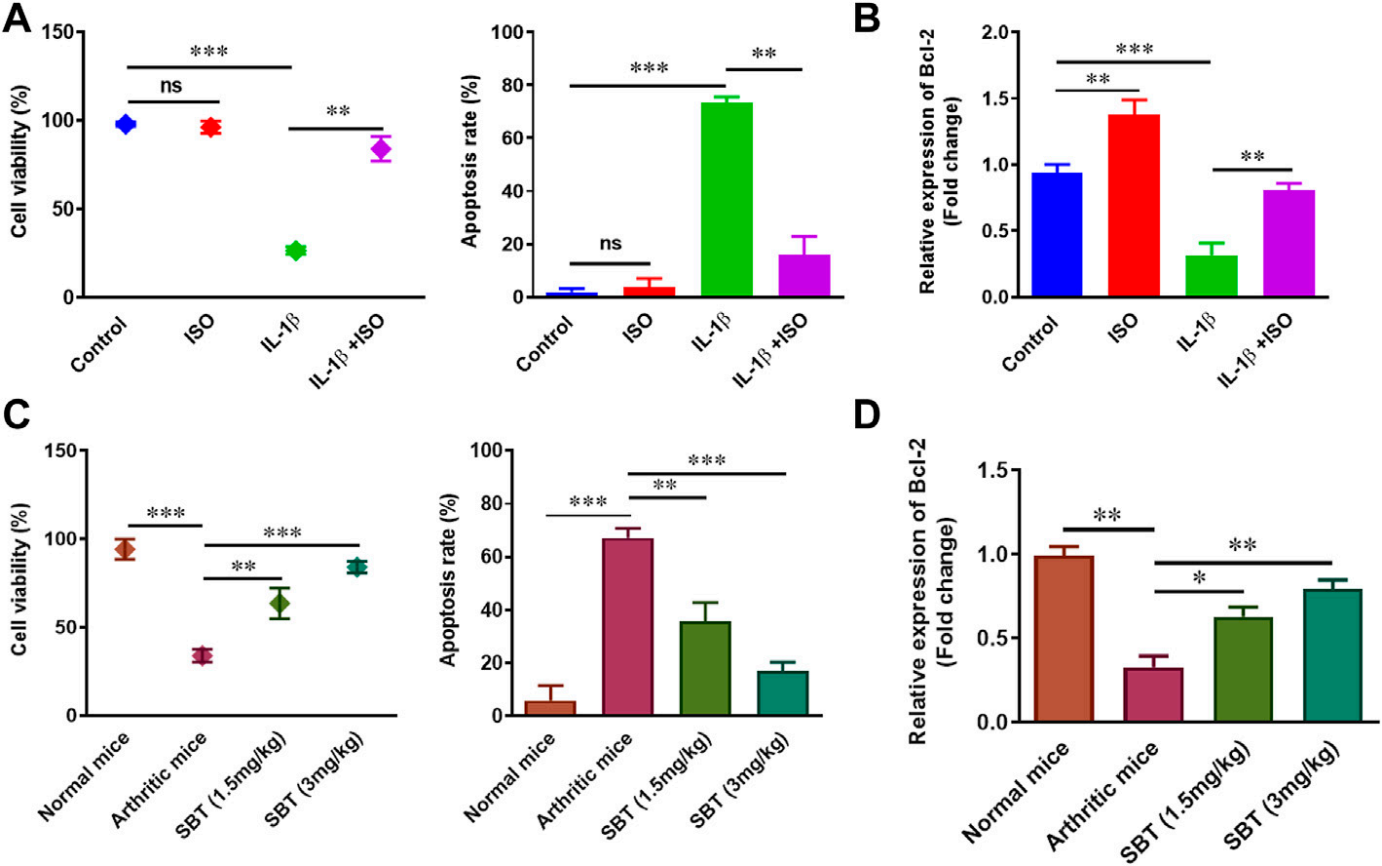
Cell survival and apoptosis analysis *in vitro* and *in vivo*. 1 × 10^5^ CHON-001 cells were seeded in complete DMEM culture medium. **(A)** Cell viability and apoptosis of CHON-001 cells was assessed by Trypan blue exclusion method. **(B)** Relative *Bcl-2* expression in CHON-001 cells measured by using qPCR. Results were analyzed by 2^–∆∆Ct^ method. **(C)** Cell viability and apoptosis rate of murine chondrocytes were assessed by Trypan Blue exclusion method. **(D)** qPCR analysis of relative *Bcl-2* expression in murine chondrocytes. All experiments were performed in duplicates. Data were analyzed by GraphPad prism version 7.0. Significance of data was measured by using One Way ANOVA. *p < 0.05, **p < 0.01, ***p < 0.001, ns, not significant.

Next, we wanted to examine the effect of β2-AR on cell viability and apoptosis rate in chondrocytes. IL-1β (10 ng/ml) led to a substantial decline in cellular survival and enhanced apoptosis. Isoprenaline (ISO, 10^−5^ M) did not affect these parameters in resting stage, but ISO reversed the effects of IL-1β and restored the survival of CHON-001 cells (Figure 3A). As we know, Bcl-2 is an anti-apoptotic protein often used as a marker to assess cellular apoptosis rate (Chipuk et al., 2008; Bano et al., 2022). Incubation of CHON-001 cells with ISO significantly upregulated *Bcl-2* gene expression (Figure 3B). SBT (1.5 mg/kg and 3 mg/kg) increased cell survival and Bcl-2 expression while decreased apoptosis in a dose-dependent manner in murine chondrocytes (Figures 3C,D). Overall, our results demonstrated that β2-AR can improve cellular survival in chondrocytes by decreasing apoptosis in arthritis.

### ISO and SBT inhibit pro-inflammatory cytokine production in arthritic chondrocytes

**FIGURE 4:**
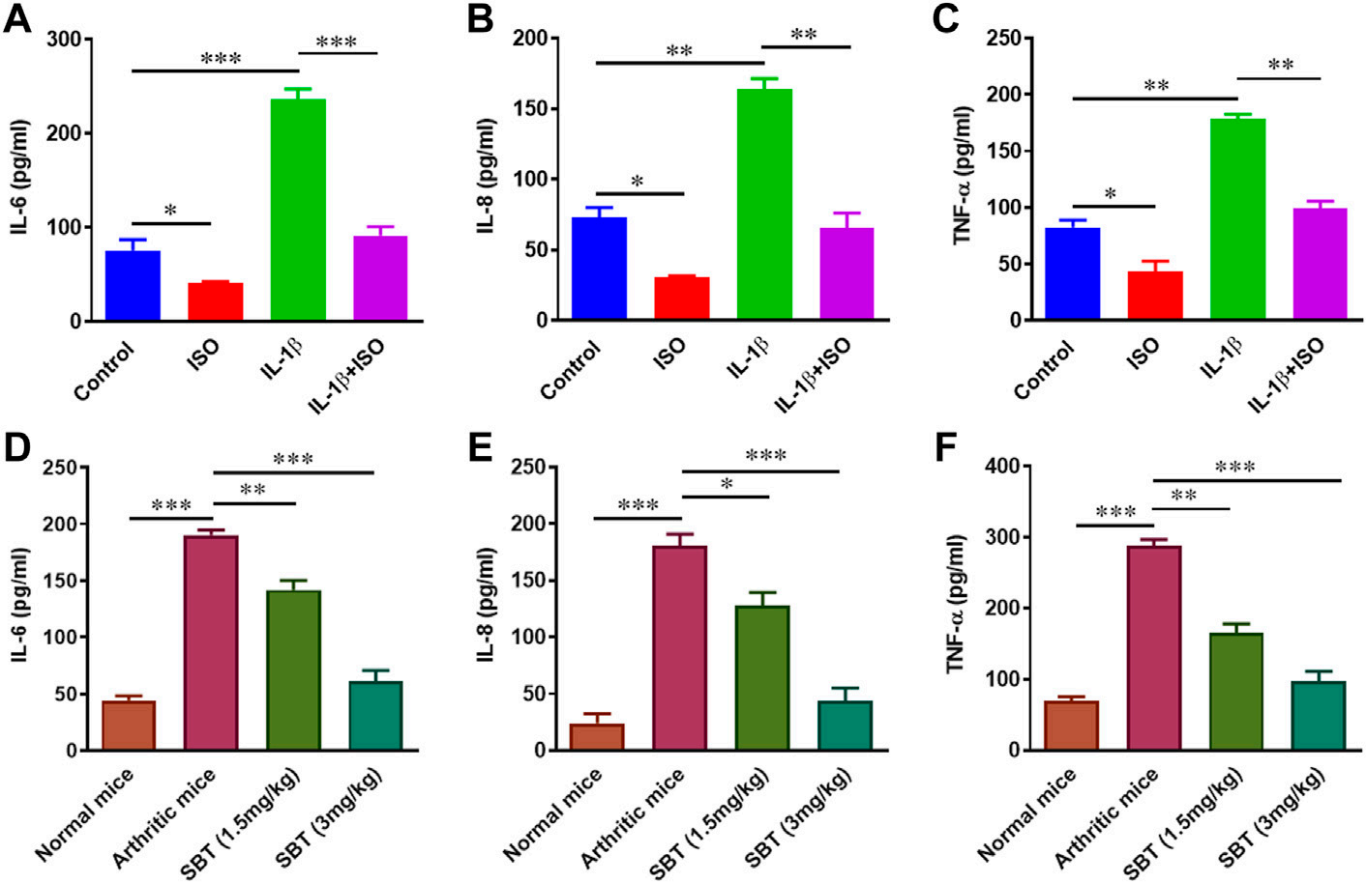
Protein quantification analysis of pro-inflammatory cytokines *in vitro* and *in vivo*. CHON-001 cells were treated with ISO (10^−5 ^mol/L), IL-1β (10 ng/ml) or combination of both for 24 h and cell supernatants were collected. Protein levels ofIL-6 **(A)**, IL-8 **(B)** and TNF-α **(C)** were analyzed by ELISA. Articular chondrocytes were cultured in complete DMEM medium for 24 h. After incubation, cell supernatants were collected and ELISA was performed to quantify IL-6 **(D)**, IL-8 **(E)** and TNF-α **(F)** at protein levels in murine chondrocytes. Pro-Quantum software was used to analyze the results. All experiments were performed in duplicates. Significance of data was measured by using One Way ANOVA. *p < 0.05, **p < 0.01, ***p < 0.001.

During the course of arthritis, pro-inflammatory cytokines play critical role in synovial inflammation and joint destruction (Schuerwegh et al., 2003; Hassan et al., 2022). So next we wanted to sort out whether or not β2-AR can impact the production of cytokines in chondrocytes. ELISA analysis for the protein quantification of IL-6, IL-8 and TNF-α indicated that IL-1β enhanced the production of these cytokines. ISO (10^−5 ^mol/L) restored IL-1β-induced rise in IL-6, IL-8 and TNF-α protein levels (Figures 4A–C). Similar results were obtained in chondrocytes from arthritic mice, where SBT- treated mice had lower production of these cytokines in a dose-dependent manner (Figures 4D–F). These results indicate that β2-AR agonist can significantly downregulate the production of pro-inflammatory cytokines in arthritic chondrocytes.

### ISO and SBT decrease oxidative stress and genotoxicity in arthritic chondrocytes

**FIGURE 5:**
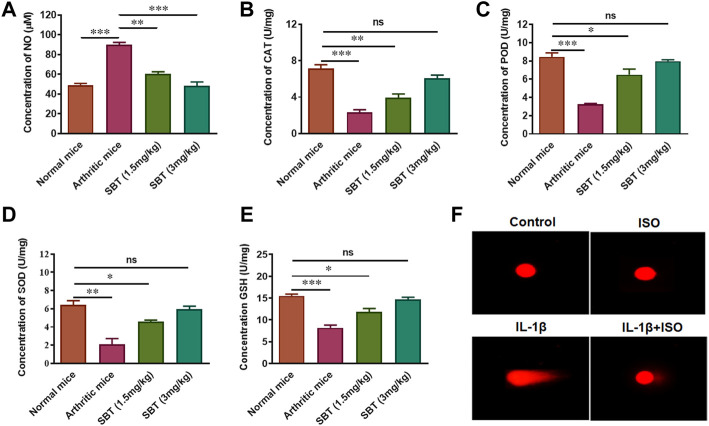
Antioxidant enzyme analysis *in vitro* and *in vivo*. Mice (*n* = 4) from each experimental group were slaughtered at day 28 and cartilage homogenates were isolated. These cartilage homogenates were subjected to centrifugation and the serum was analyzed to determine the concentration of NO **(A)**, CAT **(B)**, POD **(C)**, SOD **(D)** and GSH **(E)**. **(F)** Comet Assay. Cells were collected after 3 h’ treatment with Control, ISO, IL-1β and IL-1β+ISO and run on 1% agarose gel. The assessment of nuclear fragmentation of CHON-001 cells was performed. Images (200×) were analyzed by using CASPLAB. *p < 0.05, **p < 0.01, ***p < 0.001, ns, not significant.

Arthritis is accompanied by increased oxidative stress which leads to DNA damage (Chen et al., 2008; Neri et al., 2021). SBT-treated arthritic mice significantly decreased nitric oxide (NO) production in murine cartilage homogenates (Figure 5A). We found preserved levels of antioxidant enzymes (CAT, POD, SOD, and GSH) in cartilage of SBT-treated arthritic mice (Figures 5B–E). ISO reduced the DNA damage caused by IL-1β in CHON-001 cells, as evidenced by comet tail and other comet parameters (Figure 5F, Supplementary Table S2). Altogether, these data mean that β2-AR can decrease oxidative stress, preserve antioxidant enzymes and inhibit genotoxicity in arthritic chondrocytes.

### ISO and SBT inhibit glucose transport into arthritic chondrocytes

**FIGURE 6:**
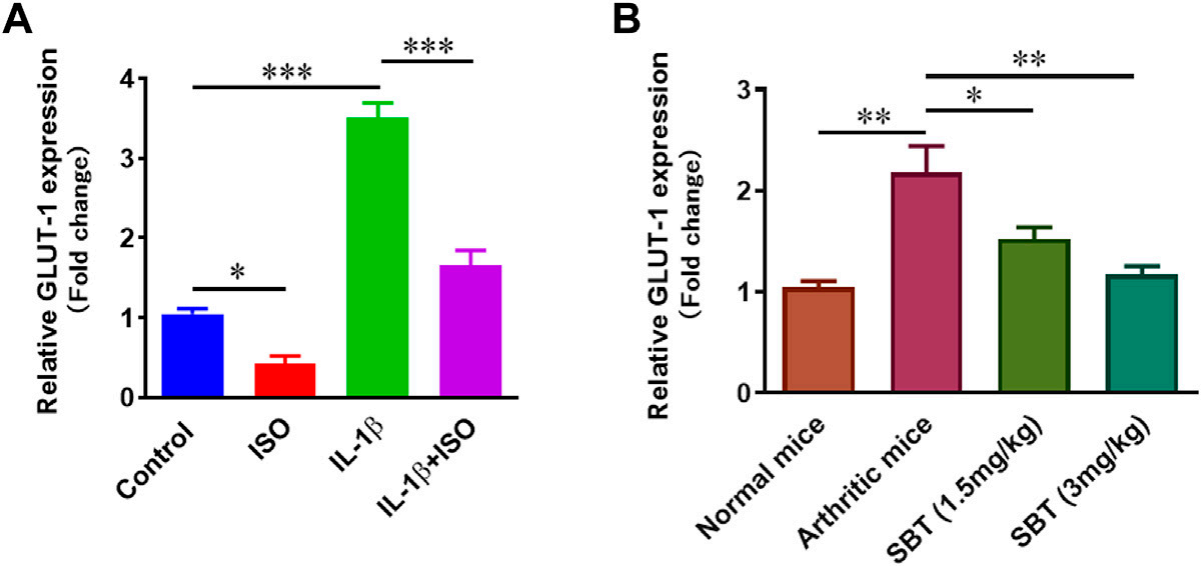
*GLUT-1* gene expression analysis *in vitro* and *in vivo*. **(A)** CHON-001 cells were treated with ISO (10^−5 ^mol/L), IL-1β (10 ng/ml) or combination of both for 24 h. *GLUT-1* gene expression was analyzed by using qPCR technique. **(B)** Articular chondrocytes were isolated from each experimental mice group at day 28 and qPCR was performed to analyze the gene expression of *GLUT-1*. GAPDH was used as a reference. All experiments were performed in duplicates. Significance of data was measured by using One Way ANOVA. *p < 0.05, **p < 0.01, ***p < 0.001.

Higher levels of glucose can cause substantial damage to chondrocytes in RA by producing excess ROS (Rosa et al., 2011). In order to determine the impact of β2-AR on transport of glucose, *GLUT-1* expression in CHON-001 cells and murine articular chondrocytes was analyzed by qPCR using respective primers for human and mice. In CHON-001 cells, we found that ISO partially inhibited IL-1β-induced increase of GLUT-1 expression signifying that β2-AR can inhibit the transport of glucose (Figure 6A). Similar results that SBT downregulated *GLUT-1* gene expression in a dose-dependent manner were obtained in murine articular chondrocytes from SBT-treated mice (Figure 6B).

### ISO and SBT promote mitochondrial biogenesis in arthritic chondrocytes

**FIGURE 7:**
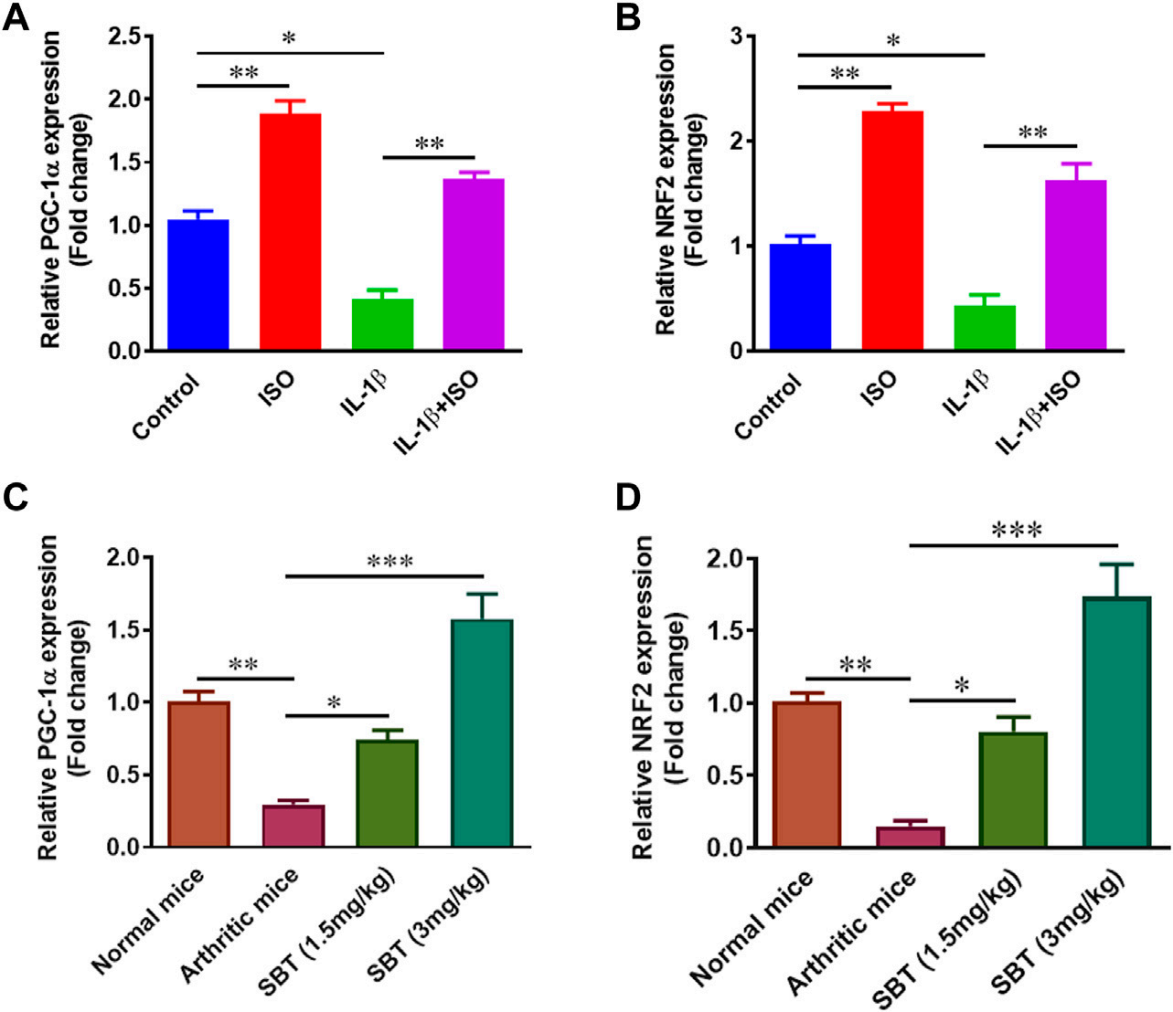
Mitochondrial biogenesis regulation analysis *in vitro* and *in vivo*. CHON-001 cells were treated with ISO (10^−5 ^mol/L), IL-1β (10 ng/ml) or combination of both for 24 h. Quantitative-PCR was performed to analyze relative expression of *PGC-1α* **(A)** and *NRF2* **(B)**. Relative gene expression analysis of *PGC-1α* **(C)** and *NRF2* **(D)** in murine chondrocyte by using qPCR. All experiments were performed in duplicates. Data were analyzed by GraphPad prism version 7.0. Significance of data was measured by using One Way ANOVA. *p < 0.05, **p < 0.01, ***p < 0.001.

Mitochondrial biogenesis plays key role in normal chondrocyte function and PGC-1α and NRF2 genes are important regulators of mitochondrial biogenesis (Fernandez-Marcos and Auwerx, 2011; Gureev et al., 2019). Gene expression analysis for *PGC-1α* and *NRF2* was carried out in CHON-001 cells and murine chondrocytes by using qPCR. ISO not only increased *PGC-1α* and *NRF2* in resting stage but also recovered IL-1β-mediated downregulation of these genes *in vitro* (Figures 7A,B). Our quantitative-PCR analysis demonstrated that *PGC-1α* and *NRF2* was significantly upregulated in SBT-treated mice in a dose-dependent manner (Figures 7C,D). Altogether, these data indicate that chondrocytes from arthritic mice models present deficiency in mitochondrial biogenesis, whereas β2-AR agonist can boost mitochondrial biogenesis by elevating the mitochondrial biogenesis regulator molecules.

### ISO and SBT impede matrix degradation in arthritic chondrocytes

**FIGURE 8:**
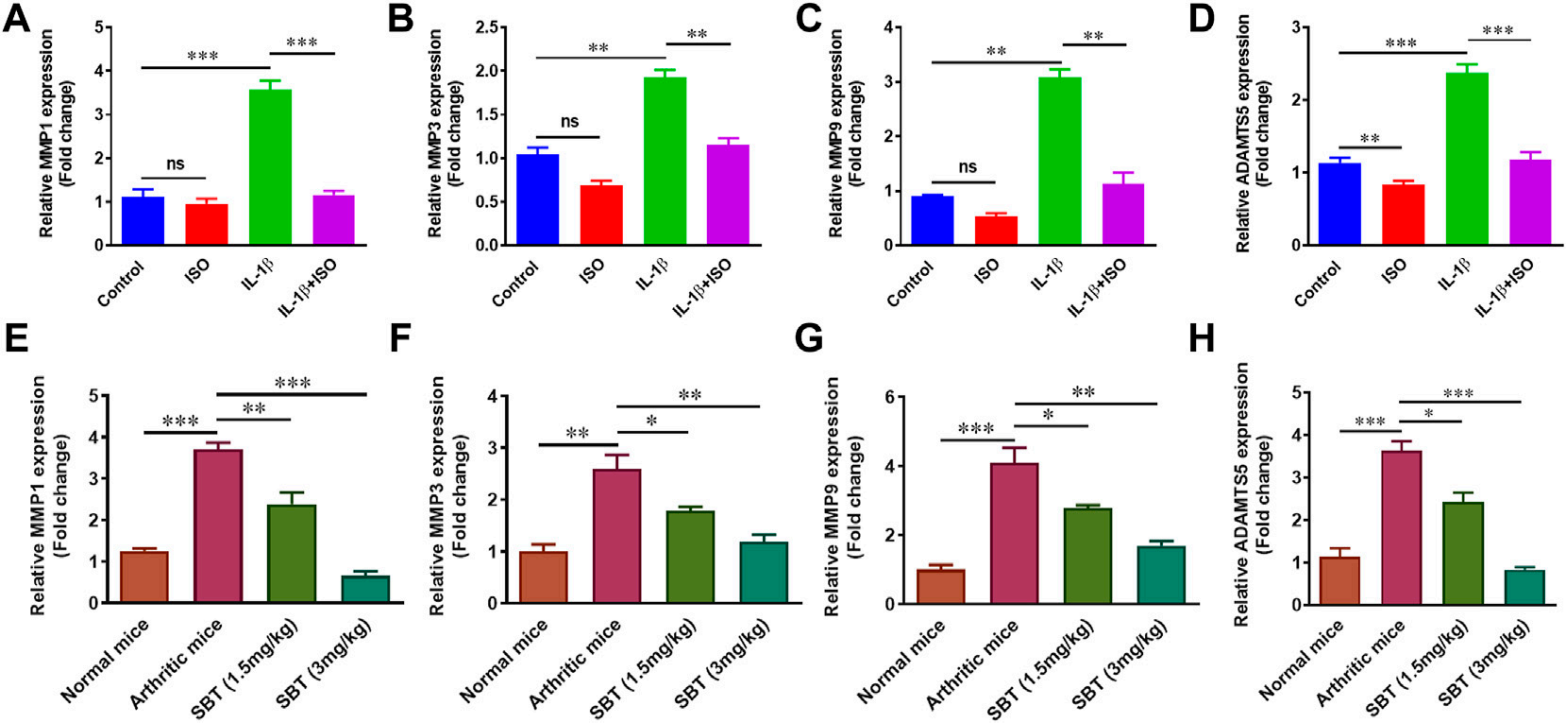
Gene expression analysis of Matrix degrading enzymes *in vitro* and *in vivo*. CHON-001 cells were treated with ISO (10^−5 ^mol/L), IL-1β (10 ng/ml) or combination of both for 24 h. Total RNAs were extracted to perform the qPCR for *MMP1* **(A)**, *MMP3* **(B)**, *MMP* **(C)** and *ADAMTS5* **(D)**. Mice from each experimental group was euthanized on 28^th^ day. Murine chondrocytes were isolated to measure the gene expression level of *MMP1* **(E)**, *MMP3* **(F)**, MMP9 **(G)** and *ADAMTS5* **(H)** by using qPCR technique. All the experiments were performed in duplicate manner. Data were analyzed by Graph-Pad prism version 7.0. Significance of data was measured by using One Way ANOVA. *p < 0.05, **p < 0.01, ***p < 0.001, ns, not significant.

In RA, chondrocytes play dual role *i.e.,* acting both as target cells as well as effector cells. Chondrocytes are directly and indirectly involved in matrix degradation by releasing certain enzymes (Baker et al., 2018). IL-1β (10 ng/ml) significantly upregulated the marker molecules such as MMP1, MMP3, MMP9 and ADAMTS5 *in vitro*. Interestingly, ISO did not affect gene expression of *MMP1*, *MMP3* and *MMP9* in resting condition but reversed the effect of IL-1β on arthritic chondrocytes (Figures 8A–D). Similar pattern was observed in mice models. qPCR analysis of murine chondrocytes indicated that administration of SBT led to a decline in *MMP1, MMP3, MMP9* and *ADAMTS5* gene expression (Figures 8E–H). Our results demonstrate that β2-AR can halt matrix degradation in arthritis by downregulating matrix degrading enzymes in chondrocytes.

### ISO and SBT promote proteoglycan synthesis in arthritic chondrocytes

**FIGURE 9:**
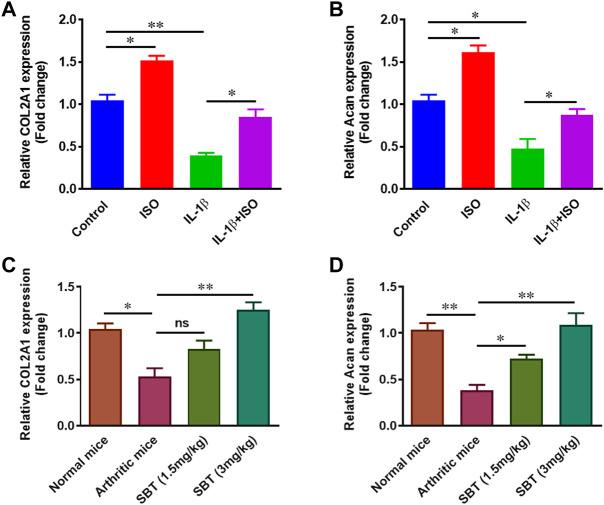
Gene expression analysis of proteoglycans *in vitro* and *in vivo*. CHON-001 cells were treated with ISO (10^−5 ^mol/L), IL-1β (10 ng/ml) or combination of both for 24 h. Gene expression analysis of *COL2A1* **(A)** and *Acan* **(B)** was measured by using qPCR technique. Relative gene expression analysis of *COL2A1* **(C)** and *Acan* **(D)** in murine chondrocytes by using qPCR. All the experiments were performed in duplicate manner. Data were analyzed by Graph-Pad prism version 7.0. Significance of data was measured by using One Way ANOVA with Boneferroni’s multiple comparison test. *p < 0.05, **p < 0.01, ns, not significant.

IL-1β downregulated the synthesis of proteoglycan in chondrocytes as reported previously (Kato et al., 2014). Here, we found ISO increased the expression of *COL2A1* and *Acan* in resting stage and inhibited IL-β-mediated downregulation of these genes in CHON-001 cells (Figures 9A,B). Further, in order to evaluate whether or not β2-AR can affect proteoglycan synthesis *in vivo*, chondrocytes were isolated from normal, arthritic and salbutamol-treated arthritic mice and subjected to quantitative-PCR analysis. The results showed that SBT (3 mg/kg) significantly upregulated the expression of both *COL2A1* and *Acan* genes *in vivo* (Figures 9C,D). Collectively, these data demonstrate that β2-AR agonist significantly increased the expression of genes involved in proteoglycan synthesis.

### β2-AR agonist conserves β2-AR gene expression in arthritic chondrocytes

**FIGURE 10:**
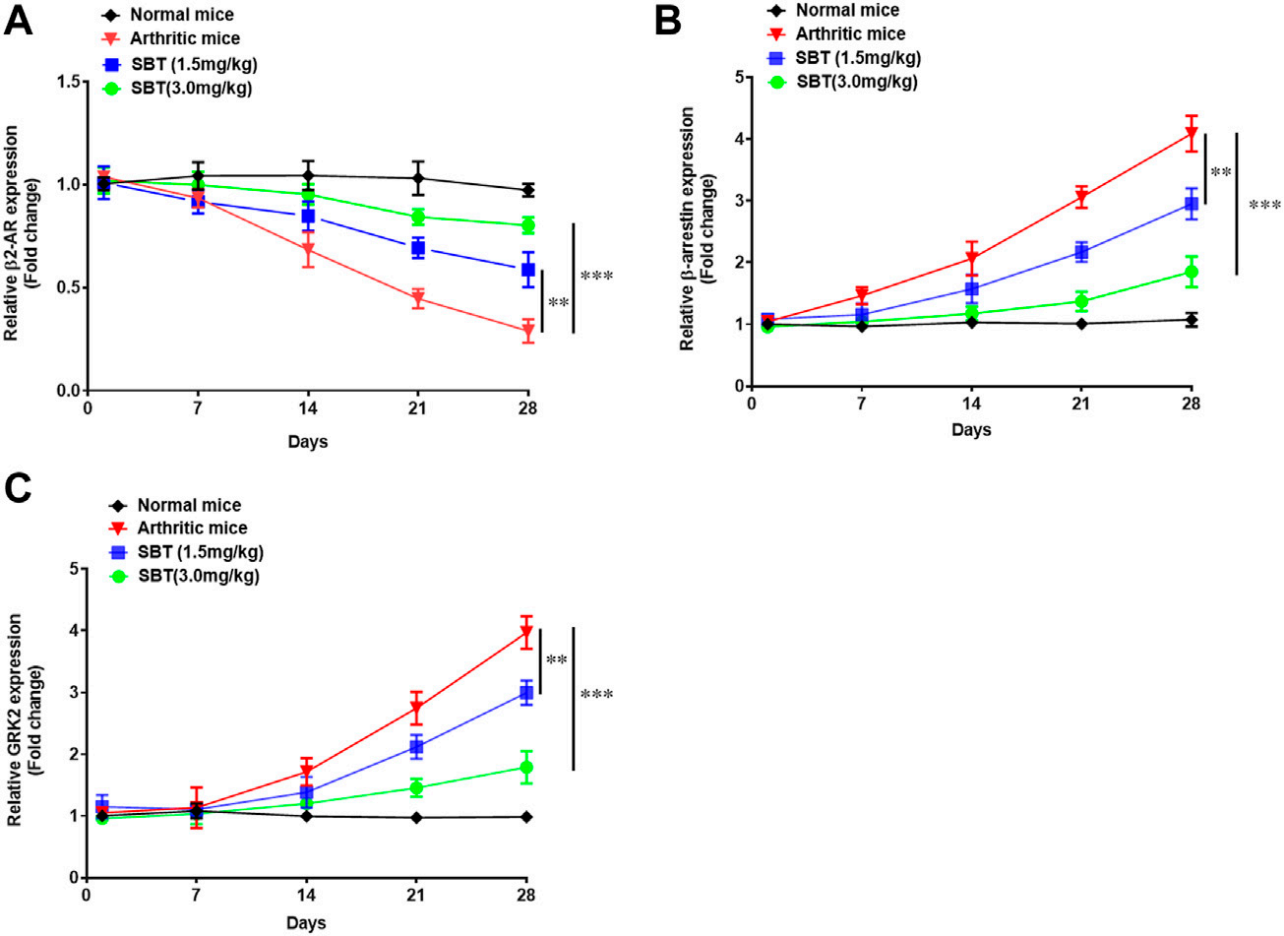
Mice were euthanized on weekly basis under 4% chloral hydrate general anesthesia, and chondrocytes were isolated from mice cartilage under sterile conditions. RNAs were isolated by Trizol reagent, converted to cDNA and qPCR was performed to analyze the relative expression of *β2-AR* **(A)**, *β-arrestin* **(B)** and *GRK2* **(C)**. Data were analyzed by GraphPad prism version 7.0. Significance of data was measured by using Two Way ANOVA with Boneferroni’s multiple comparison test. **p < 0.01, ***p < 0.001.

Next, we wanted to find out how arthritis does affect the expression of β2-AR in mice chondrocytes. Briefly, mice from each group (n = 2) were euthanized on weekly basis at 1^st^, 7^th^, 14^th^, 21^st^ and 28^th^ day post immunization and chondrocytes were isolated. In CFA-treated mice, there was no difference in β2-AR expression till disease onset (day 14). At 21^st^ and 28^th^ day post immunization, β2-AR expression in chondrocytes was significantly decreased (Figure 10A), which was consistent with the data in Figure 1F. While SBT (1.5 mg/kg and 3 mg/kg) significantly inhibited the downregulation of β2-AR expression in arthritic mice when compared with arthritic control (Figure 10A). Similarly, SBT significantly decreased arthritis- induced upregulation of β-arrestin and GRK2 in murine chondrocytes (Figures 10B,C). The data demonstrate that β2-AR agonist administered at disease onset can inhibit receptor internalization by downregulating the expression of *β-arrestin* and *GRK2* in chondrocytes.

## Discussion

Inflammation of synovium marks the major hallmark of arthritis and there is a gradual loss of cartilage and bones (Maeda et al., 2022; Sun et al., 2022). The exact pathogenic process of RA remains still a mystery, and various pathological changes are proposed to be the causative agents of disease (Cutolo and Straub, 2006; McInnes and Schett, 2007; McInnes and Schett, 2011; Xue et al., 2022). Since the last decade or so, there has been extensive research into the involvement of the sympathetic nervous system in immunity, and as a result, the neuroimmune nexus has become a hot issue in tumor immunology and inflammatory illnesses (Lorton and Bellinger, 2015; Qiao et al., 2018; Jensen et al., 2021). β2-AR downregulates various pro-inflammatory pathways by inhibiting the activity of immune cells. Whereas, β2-AR can inhibit the activation and proliferation of cytotoxic T lymphocytes, and helper T cells which involve in autoimmune diseases (Landmann, 1992; Sanders and Straub, 2002; Wu et al., 2018). Moreover, dendritic cell maturation and antigen presenting ability are also affected negatively by β2-AR signaling (Nijhuis et al., 2014).

In RA, β2-AR signaling is dysregulated characterized by downregulation of β2-AR receptor and hence response of T lymphocytes to sympathomimetic drugs is hampered. Higher epinephrine levels have been reported in patient with arthritis, and epinephrine has been shown to worsen arthritis symptoms in mice models via its action on sympathetic efferent nerve terminals (Coderre et al., 1990; Vlcek et al., 2008). Activation of sympathetic nervous system, and exposure of β2-AR to high concentrations of epinephrine in arthritis leads to low levels of cAMP production by β2-AR hence receptor desensitization and downregulation in splenocytes and lymph nodes of CFA mice model takes place. Loss of receptor density in mice was consistent with an increase in disease severity (Lorton and Bellinger, 2015). Previous research about the role of β2-AR signaling in arthritis revolves around its expression patterns in different immune cells and cells of vital immune organs like spleen and lymph nodes, and detailed analysis of β2-AR on chondrocyte function has not been reported yet.

In present study, gene expression analysis of β2-AR in chondrocytes revealed downregulated pattern in both *in vitro* and *in vivo* disease models. In CHON-001 cells, IL-1β led to a downregulation of β2-AR receptors. Our *in vivo* results mimicked *in vitro* findings (Figure 1). That is, why β2-AR receptor agonist SBT was used in CFA mice model, and SBT (1.5 mg/kg and 3 mg/kg) significantly attenuated the disease as shown in Figure 2. So detailed analysis of the role of β2-AR in chondrocytes was carried out in further experiments. RA and OA are characterized by progressive degeneration of cartilage and chondrocyte apoptosis. In CHON-001 cells, ISO inhibited IL-1β-induced apoptosis as well as preserved cell viability. Moreover, IL-1β-induced loss of *Bcl-2* expression was also restored by β2-AR agonist ISO. In CFA mice model, SBT significantly increased cell viability and inhibited apoptosis of the isolated chondrocytes, which was consistent with the *in vitro* data.

The production of pro-inflammatory cytokines and the loss of mitochondrial biogenesis in chondrocytes cause cellular death and other procatabolic reactions (Kan et al., 2021). Macrophages and synovial fibroblasts stimulate chondrocytes and as a result TNF-α is released. TNF-α further stimulates the production of IL-6 and IL-8 (Feng et al., 2016). In ELISA analysis for pro-inflammatory cytokine production, it was evident that β2-AR agonist leads to production of lesser amounts of IL-6, IL-8 and TNF-α in chondrocytes of arthritic mice and IL-1β-treated human chondrocyte cell line CHON-001. Cytokine production by chondrocytes further complicates the picture and results in oxidative damage by increased production of reactive oxygen species and reactive nitrogen species. In current study, NO production in murine articular cartilage was decreased by the use of SBT and a rise in the level of antioxidant enzymes CAT, POD, SOD and GSH was observed. Rise in endogenous antioxidant enzymes in chondrocytes is advantageous due to the fact that free radicals have notorious property of extracellular matrix degradation as well as tissue damage. This cytokine-mediated matrix degradation in RA is mainly caused by lipid peroxidation and can be prevented in cytokine-stimulated chondrocytes by the addition of antioxidant enzymes (Tiku et al., 2000; Ferreira et al., 2021). Decrease in IL-1β-induced DNA damage by ISO in CHON-001 cells might owe to their property to decrease oxidative stress *in vivo* by downregulating GLUT-1 gene expression in chondrocytes. Previously β2-AR has been reported to inhibit *GLUT-1* expression in astrocytes, adipocytes as well as in T-lymphocytes (Mulder et al., 2005; Dong et al., 2012; Qiao et al., 2019).

Dysregulation of mitochondrial biogenesis is considered as a hallmark of joint pathology. Deficiency of mitochondrial biogenesis leads to an increase in procatabolic signaling in chondrocytes (Mignotte et al., 1991). ISO and SBT led to an increase in mitochondrial biogenesis of chondrocytes by upregulating PGC-1α and NRF2 both *in vitro and in vivo*. Recent data have demonstrated that loss of mitochondrial function in RA can be reversed by upregulation of PGC-1α, the master regulator of mitochondrial metabolism (Wang and Hekimi, 2015). Increased PGC-1α expression in chondrocytes by β2-AR agonist could be explained by the fact that activation of β2-AR leads to activation of PKA which can activate CREB elements downstream which further stimulates PGC-1α expression (Boss et al., 1999). The role of PGC-1α in mitochondrial biogenesis is well established but it is NRF2 that has recently emerged as an important player in the said phenomenon. Deletion of *NRF2* in animal models led to no change in markers of mitochondrial fitness by *NRF2* activators (Chen et al., 2014). NRF2 also activates the release of antioxidant enzymes in PGC-1α-dependent manner, and deletion of PGC-1α resulted in failure to release of NRF2-dependent SOD (Lee et al., 2020). So the fact that β2-AR can increase anti-oxidant enzymes in murine chondrocytes can be correlated with its property to increase *PGC-1α* and *NRF2* gene expression.

Articular cartilage lacks blood vessels and mainly comprises of chondrocytes and extracellular matrix (ECM). Chondrocytes are embedded into ECM and in RA, it is the chondrocytes that release matrix-degrading enzymes responsible for ECM destruction (Mauck et al., 2007). Extracellular matrix degradation takes place when articular chondrocytes release MMPs. β2-AR signaling in RA, significantly inhibited the expression of MMP1, MMP3 and MMP9 in salbutamol-treated mice when compared with arthritic control, and the similar results was observed in CHON-001 cells. There are about 26 different types of MMPs but levels of MMP1, MMP3 and MMP9 are specifically elevated in RA leading to matrix degradation (Ni et al., 2019). Apart from MMPs, ADAMTSs are another class of enzymes involved in matrix degradation. Quantitative-PCR analysis of *ADAMTS5* gene expression indicated a declining trend in chondrocytes treated by ISO or IL-1β+ISO when compared with normal and IL-1β-treated chondrocytes, respectively. β2-AR mediated downregulation of *MMPs* and *ADAMTS5* genes in chondrocytes could be a positive sign for matrix protection in arthritis. Apart from reduction of matrix degrading markers, β2-AR agonists (SBT and ISO) led to an increase in genes involved in proteoglycan synthesis. RT-qPCR analysis indicated that β2-AR agonists can upregulate expression of *Acan* and *COL2A1* involved in the biosynthesis of cartilage *in vitro* and *in vivo*.

Previously isoproterenol has been studied for its role in embryonic cartilage development in mice model. The authors demonstrated that β2-AR is involved in the process of bone mineralization via collagen type Ⅹ and indian hadgehog through ERK1/2 pathway (Lai and Mitchell, 2008). In our arthritis model, we found an increased expression of β-arrestin and GRK2 in chondrocytes, but β2-AR agonist when administered at disease onset throughout the course of study, inhibited β-arrestin and GRK2 expression (Figure 10). Our results are in agreement with Wu H et al., 2019, where authors demonstrated in fibroblast synoviocytes, that desensitization of β2-AR and increased expression of GRKs in arthritis is relevant to augmented inflammation in arthritis model (Wu et al., 2019). Our findings suggest that β2-AR agonists used in the early course of arthritis could inhibit inflammation and prevent receptor desensitization by β-arrestin and GRK2 signaling in chondrocytes.

## Conclusion

β2-AR agonists are effective at treating RA symptoms as well as preventing problems like cartilage degradation by preserving chondrocyte cells. β2-AR receptor agonists can prevent detrimental chondrocyte alterations brought on by ROS and support cartilage hemostasis. Therefore, including ISO or SBT in clinical settings may be advantageous, but this notion is still too premature, and more research is needed to clarify the role of β2-AR in chondrocytes at the pharmacological and molecular levels.

## Data Availability

The original contributions presented in the study are included in the article/Supplementary Materials, further inquiries can be directed to the corresponding authors.

## Abbreviations

RA, Rheumatoid arthritis; IL-1β, Interleukin-1 beta; β2-AR, β2-adrenergic receptor; MTX, Methotrexate; SBT, Salbutamol; ISO, Isoprenaline; Bcl-2, B- cell lymphoma 2; TNF-α, Tumor necrosis factor alpha; IL-6, Interleukin-6; IL-8, Interleukin-8; ELISA, Enzyme-linked immunosorbent assay; NO, Nitric Oxide; CAT, Catalase; POD, Peroxidase; SOD, Superoxide dismutase; GSH, Glutathione; MMPs, Matrix Metalloproteinase; ADAMTS5, ADAM Metallopeptidase With Thrombospondin Type 1 Motif 5; GLUT-1, Glucose Transporter type 1; PGC-1α, Peroxisome proliferator-activated receptor gamma coactivator 1-alpha; NRF2, Nuclear factor erythroid 2-related factor 2; COL2A1, Collagen Type 2 alpha 1 chain; Acan, Aggrecan.

## References

[B1] Aletaha D. Smolen J. S. (2018). Diagnosis and management of rheumatoid arthritis: A review. Jama 320 (13), 1360–1372. 10.1001/jama.2018.13103 30285183

[B2] Baker J. Falconer A. M. Wilkinson D. J. Europe-Finner G. N. Litherland G. J. Rowan A. D. (2018). Protein kinase D3 modulates MMP1 and MMP13 expression in human chondrocytes. PLoS One 13 (4), e0195864. 10.1371/journal.pone.0195864 29652915 PMC5898748

[B3] Bano I. Horky P. Abbas S. Q. Majid M. Bilal A. H. M. Ali F. (2022). Ferroptosis: A new road towards cancer management. Molecules 27 (7), 2129. 10.3390/molecules27072129 35408533 PMC9000380

[B4] Bellinger D. L. Wood C. Wergedal J. E. Lorton D. (2021). Driving β2-while suppressing α-adrenergic receptor activity suppresses joint pathology in inflammatory arthritis. Front. Immunol. 12, 628065. 10.3389/fimmu.2021.628065 34220796 PMC8249812

[B5] Boss O. Bachman E. Vidal-Puig A. Zhang C. Y. Peroni O. Lowell B. B. (1999). Role of the beta(3)-adrenergic receptor and/or a putative beta(4)-adrenergic receptor on the expression of uncoupling proteins and peroxisome proliferator-activated receptor-gamma coactivator-1. Biochem. Biophys. Res. Commun. 261 (3), 870–876. 10.1006/bbrc.1999.1145 10441518

[B6] Chen A. F. Davies C. M. De Lin M. Fermor B. (2008). Oxidative DNA damage in osteoarthritic porcine articular cartilage. J. Cell. Physiol. 217 (3), 828–833. 10.1002/jcp.21562 18720406 PMC2575799

[B7] Chen H. Hu Y. Fang Y. Djukic Z. Yamamoto M. Shaheen N. J. (2014). Nrf2 deficiency impairs the barrier function of mouse oesophageal epithelium. Gut 63 (5), 711–719. 10.1136/gutjnl-2012-303731 23676441 PMC3883925

[B8] Chipuk J. E. Fisher J. C. Dillon C. P. Kriwacki R. W. Kuwana T. Green D. R. (2008). Mechanism of apoptosis induction by inhibition of the anti-apoptotic BCL-2 proteins. Proc. Natl. Acad. Sci. U. S. A. 105 (51), 20327–20332. 10.1073/pnas.0808036105 19074266 PMC2629294

[B9] Coderre T. J. Basbaum A. I. Dallman M. F. Helms C. Levine J. D. (1990). Epinephrine exacerbates arthritis by an action at presynaptic B2-adrenoceptors. Neuroscience 34 (2), 521–523. 10.1016/0306-4522(90)90160-6 2159131

[B10] Cutolo M. Straub R. H. (2006). Stress as a risk factor in the pathogenesis of rheumatoid arthritis. Neuroimmunomodulation 13 (5-6), 277–282. 10.1159/000104855 17709949

[B11] Dong J. H. Chen X. Cui M. Yu X. Pang Q. Sun J. P. (2012). β2-adrenergic receptor and astrocyte glucose metabolism. J. Mol. Neurosci. 48 (2), 456–463. 10.1007/s12031-012-9742-4 22399228

[B12] Feng X. Shi Y. Xu L. Peng Q. Wang F. Wang X. (2016). Modulation of IL-6 induced RANKL expression in arthritic synovium by a transcription factor SOX5. Sci. Rep. 6 (1), 32001–32010. 10.1038/srep32001 27550416 PMC4994074

[B13] Fernandez-Marcos P. J. Auwerx J. (2011). Regulation of PGC-1α, a nodal regulator of mitochondrial biogenesis. Am. J. Clin. Nutr. 93 (4), 884S–890S. 10.3945/ajcn.110.001917 21289221 PMC3057551

[B14] Ferreira H. B. Melo T. Paiva A. Domingues M. D. R. (2021). Insights in the role of lipids, oxidative stress and inflammation in rheumatoid arthritis unveiled by new trends in lipidomic investigations. Antioxidants 10 (1), 45. 10.3390/antiox10010045 PMC7824304 33401700

[B15] Gosset M. Berenbaum F. Thirion S. Jacques C. (2008). Primary culture and phenotyping of murine chondrocytes. Nat. Protoc. 3 (8), 1253–1260. 10.1038/nprot.2008.95 18714293

[B16] Gureev A. P. Shaforostova E. A. Popov V. N. (2019). Regulation of mitochondrial biogenesis as a way for active longevity: interaction between the Nrf2 and PGC-1α signaling pathways. Front. Genet. 10, 435. 10.3389/fgene.2019.00435 31139208 PMC6527603

[B17] Hassan S. S. U. Muhammad I. Abbas S. Q. Hassan M. Majid M. Jin H. Z. (2021). Stress driven discovery of natural products from actinobacteria with anti-oxidant and cytotoxic activities including docking and admet properties. Int. J. Mol. Sci. 22 (21), 11432. 10.3390/ijms222111432 34768863 PMC8584265

[B18] Hassan S. S. U. Zhang W. D. Jin H. Z. Basha S. H. Priya S. S. (2022). *In-silico* anti-inflammatory potential of guaiane dimers from Xylopia vielana targeting COX-2. J. Biomol. Struct. Dyn. 40 (1), 484–498. 10.1080/07391102.2020.1815579 32876526

[B19] Jensen A. W. P. Carnaz Simões A. M. thor Straten P. Holmen Olofsson G. (2021). Adrenergic signaling in immunotherapy of cancer: friend or foe? Cancers 13 (3), 394. 10.3390/cancers13030394 33494360 PMC7866099

[B20] Kan S. Duan M. Liu Y. Wang C. Xie J. (2021)., 13. Cartilage, 1102S–1121S. 10.1177/19476035211063858 Role of mitochondria in physiology of chondrocytes and diseases of osteoarthritis and rheumatoid arthritis Cartilage 34894777 PMC8804744

[B21] Kato T. Miyaki S. Ishitobi H. Nakamura Y. Nakasa T. Lotz M. K. (2014). Exosomes from IL-1β stimulated synovial fibroblasts induce osteoarthritic changes in articular chondrocytes. Arthritis Res. Ther. 16 (4), 1633–R211. 10.1186/ar4679 PMC4261911 25092378

[B22] Lai L. P. Mitchell J. (2008). Beta2-adrenergic receptors expressed on murine chondrocytes stimulate cellular growth and inhibit the expression of Indian hedgehog and collagen type X. J. Cell. Biochem. 104 (2), 545–553. 10.1002/jcb.21646 18059015

[B23] Landmann R. (1992). Beta-adrenergic receptors in human leukocyte subpopulations. Eur. J. Clin. Invest. 22, 30–36. PMID: 1333965. 1333965

[B24] Lee S. Y. Wu S. T. Su M. J. Liang Y. J. Ku H. C., 2020. Dipeptidyl peptidase-4 involved in regulating mitochondria function in cardiomyocytes through Nrf2 and PGC-1α signaling. DOI: 10.21203/rs.3.rs-30722/v1

[B25] Levine J. D. Coderre T. J. Helms C. Basbaum A. I. (1988). Beta 2-adrenergic mechanisms in experimental arthritis. Proc. Natl. Acad. Sci. U. S. A. 85 (12), 4553–4556. 10.1073/pnas.85.12.4553 2837769 PMC280469

[B26] Lorton D. Bellinger D. L. (2015). Molecular mechanisms underlying β-adrenergic receptor-mediated cross-talk between sympathetic neurons and immune cells. Int. J. Mol. Sci. 16 (3), 5635–5665. 10.3390/ijms16035635 25768345 PMC4394497

[B27] Lubahn C. L. Lorton D. Schaller J. A. Sweeney S. J. Bellinger D. L. (2014). Targeting α-and β-adrenergic receptors differentially shifts Th1, Th2, and inflammatory cytokine profiles in immune organs to attenuate adjuvant arthritis. Front. Immunol. 5, 346. 10.3389/fimmu.2014.00346 25157248 PMC4127464

[B28] Maeda K. Yoshida K. Nishizawa T. Otani K. Yamashita Y. Okabe H. (2022). Inflammation and bone metabolism in rheumatoid arthritis: Molecular mechanisms of joint destruction and pharmacological treatments. Int. J. Mol. Sci. 23 (5), 2871. 10.3390/ijms23052871 35270012 PMC8911191

[B29] Mahmood F. Khan J. A. Mahnashi M. H. Jan M. S. Javed M. A. Rashid U. (2022). Anti-inflammatory, analgesic and antioxidant potential of new (2 S, 3 S)-2-(4-isopropylbenzyl)-2-methyl-4-nitro-3-phenylbutanals and their corresponding carboxylic acids through *in vitro*, in silico and *in vivo* studies. Molecules 27 (13), 4068. 10.3390/molecules27134068 35807316 PMC9268591

[B30] Majid M. Farhan A. Imran Asad M. Khan M. R. Hassan S. S. U. Haq I. U. (2022). An extensive pharmacological evaluation of new anti-cancer triterpenoid (nummularic acid) from *Ipomoea batatas* through *in vitro*, in silico, and *in vivo* studies. Molecules 27, 2474. 10.3390/molecules27082474 35458672 PMC9030838

[B31] Mauck R. L. Byers B. A. Yuan X. Tuan R. S. (2007). Regulation of cartilaginous ECM gene transcription by chondrocytes and MSCs in 3D culture in response to dynamic loading. Biomech. Model. Mechanobiol. 6 (1), 113–125. 10.1007/s10237-006-0042-1 16691412

[B32] McInnes I. B. Schett G. (2007). Cytokines in the pathogenesis of rheumatoid arthritis. Nat. Rev. Immunol. 7 (6), 429–442. 10.1038/nri2094 17525752

[B33] McInnes I. B. Schett G. (2011). The pathogenesis of rheumatoid arthritis. N. Engl. J. Med. 365 (23), 2205–2219. 10.1056/NEJMra1004965 22150039

[B34] Mignotte F. Champagne A. M. Froger-Gaillard B. Benel L. Gueride M. Adolphe M. (1991). Mitochondrial biogenesis in rabbit articular chondrocytes transferred to culture. Biol. Cell 71 (1-2), 67–72. 10.1016/0248-4900(91)90052-O 1912949

[B35] Mulder A. H. Tack C. J. Olthaar A. J. Smits P. Sweep F. C. Bosch R. R. (2005). Adrenergic receptor stimulation attenuates insulin-stimulated glucose uptake in 3T3-L1 adipocytes by inhibiting GLUT4 translocation. Am. J. Physiol. Endocrinol. Metab. 289 (4), E627–E633. 10.1152/ajpendo.00079.2004 15914506

[B36] Neri S. Guidotti S. Bini C. Pelotti S. D’Adamo S. Minguzzi M. (2021). Oxidative stress-induced DNA damage and repair in primary human osteoarthritis chondrocytes: focus on IKKα and the DNA mismatch repair system. Free Radic. Biol. Med. 166, 212–225. 10.1016/j.freeradbiomed.2021.02.020 33636333

[B37] Ni S. Li C. Xu N. Liu X. Wang W. Chen W. (2019). Follistatin‐like protein 1 induction of matrix metalloproteinase 1, 3 and 13 gene expression in rheumatoid arthritis synoviocytes requires MAPK, JAK/STAT3 and NF‐κB pathways. J. Cell. Physiol. 234 (1), 454–463. 10.1002/jcp.26580 29932210

[B38] Nijhuis L. E. Olivier B. J. Dhawan S. Hilbers F. W. Boon L. Wolkers M. C. (2014). Adrenergic β2 receptor activation stimulates anti-inflammatory properties of dendritic cells *in vitro* . PloS one 9 (1), e85086. 10.1371/journal.pone.0085086 24465481 PMC3898911

[B39] Ota M. Tanaka Y. Nakagawa I. Jiang J. J. Arima Y. Kamimura D. (2020). Role of chondrocytes in the development of rheumatoid arthritis via transmembrane protein 147–mediated NF‐κB activation. Arthritis Rheumatol. 72 (6), 931–942. 10.1002/art.41182 31785076

[B40] Otero M. Goldring M. B. (2007). Cells of the synovium in rheumatoid arthritis. Chondrocytes. Arthritis Res. Ther. 9 (5), 220–313. 10.1186/ar2292 18001488 PMC2212563

[B41] Phull A. R. Majid M. Haq I. U. Khan M. R. Kim S. J. (2017). *In vitro* and *in vivo* evaluation of anti-arthritic, antioxidant efficacy of fucoidan from Undaria pinnatifida (Harvey) Suringar. Int. J. Biol. Macromol. 97, 468–480. 10.1016/j.ijbiomac.2017.01.051 28104371

[B42] Qiao G. Chen M. Bucsek M. J. Repasky E. A. Hylander B. L. (2018). Adrenergic signaling: A targetable checkpoint limiting development of the antitumor immune response. Front. Immunol. 9, 164. 10.3389/fimmu.2018.00164 29479349 PMC5812031

[B43] Qiao G. Bucsek M. J. Winder N. M. Chen M. Giridharan T. Olejniczak S. H. (2019). β-Adrenergic signaling blocks murine CD8+ T-cell metabolic reprogramming during activation: A mechanism for immunosuppression by adrenergic stress. Cancer Immunol. Immunother. 68 (1), 11–22. 10.1007/s00262-018-2243-8 30229289 PMC6326964

[B44] Reginster J. Y. (2002). The prevalence and burden of arthritis. Rheumatology 41, 3–6. 10.1093/rheumatology/41.s1.3 12173279

[B45] Robbins J. R. Thomas B. Tan L. Choy B. Arbiser J. L. Berenbaum F. (2000). Immortalized human adult articular chondrocytes maintain cartilage-specific phenotype and responses to interleukin-1beta. Arthritis Rheum. 43 (10), 2189–2201. 10.1002/1529-0131(200010)43:10<2189::AID-ANR6>3.0.CO;2-S 11037878

[B46] Rosa S. C. Rufino A. T. Judas F. M. Tenreiro C. M. Lopes M. C. Mendes A. F. (2011). Role of glucose as a modulator of anabolic and catabolic gene expression in normal and osteoarthritic human chondrocytes. J. Cell. Biochem. 112 (10), 2813–2824. 10.1002/jcb.23196 21608018

[B47] Ruscitti P. Cipriani P. Liakouli V. Carubbi F. Berardicurti O. Di Benedetto P. (2018). The emerging role of IL-1 inhibition in patients affected by rheumatoid arthritis and diabetes. Rev. Recent Clin. Trials 13 (3), 210–214. 10.2174/1574887113666180314102651 29542422

[B48] Sanders V. M. Straub R. H. (2002). Norepinephrine, the β-adrenergic receptor, and immunity. Brain Behav. Immun. 16 (4), 290–332. 10.1006/brbi.2001.0639 12096881

[B49] Schuerwegh A. J. Dombrecht E. J. Stevens W. J. Van Offel J. F. Bridts C. H. De Clerck L. S. (2003). Influence of pro-inflammatory (IL-1α, IL-6, TNF-α, IFN-γ) and anti-inflammatory (IL-4) cytokines on chondrocyte function. Osteoarthr. Cartil. 11 (9), 681–687. 10.1016/S1063-4584(03)00156-0 12954239

[B50] Sun S. Liu H. Hu Y. Wang Y. Zhao M. Yuan Y. (2022). Selection and identification of a novel ssDNA aptamer targeting human skeletal muscle. Bioact. Mat. 20, 166–178. 10.1016/j.bioactmat.2022.05.016 PMC9157180 35663338

[B51] Tang W. Wan S. Yang Z. Teschendorff A. E. Zou Q. (2018). Tumor origin detection with tissue-specific miRNA and DNA methylation markers. Bioinformatics 34 (3), 398–406. 10.1093/bioinformatics/btx622 29028927

[B52] Tiku M. L. Shah R. Allison G. T. (2000). Evidence linking chondrocyte lipid peroxidation to cartilage matrix protein degradation: possible role in cartilage aging and the pathogenesis of osteoarthritis. J. Biol. Chem. 275 (26), 20069–20076. 10.1074/jbc.M907604199 10867027

[B53] Tseng C. C. Chen Y. J. Chang W. A. Tsai W. C. Ou T. T. Wu C. C. (2020). Dual role of chondrocytes in rheumatoid arthritis: the chicken and the egg. Int. J. Mol. Sci. 21 (3), 1071. 10.3390/ijms21031071 PMC7038065 32041125

[B54] Vlcek M. Rovensky J. Blazicek P. Radikova Z. Penesova A. Kerlik J. (2008). Sympathetic nervous system response to orthostatic stress in female patients with rheumatoid arthritis. Ann. N. Y. Acad. Sci. 1148 (1), 556–561. 10.1196/annals.1410.026 19120157

[B55] Wahle M. Krause A. Kölker S. Von Wichert P. Baerwald C. G. O. (1999). Intracellular cAMP and β2‐adrenergic receptors on CD19+ lymphocytes in patients with rheumatoid arthritis. Ann. N. Y. Acad. Sci. 876 (1), 309–311. 10.1111/j.1749-6632.1999.tb07655.x 10415626

[B56] Wang Y. Hekimi S. (2015). Mitochondrial dysfunction and longevity in animals: untangling the knot. Science 350 (6265), 1204–1207. 10.1126/science.aac4357 26785479

[B57] Wu L. Tai Y. Hu S. Zhang M. Wang R. Zhou W. (2018). Bidirectional role of β2-adrenergic receptor in autoimmune diseases. Front. Pharmacol. 9, 1313. 10.3389/fphar.2018.01313 30538630 PMC6277539

[B58] Wu H. Chen J. Wang C. Liu L. Wu Y. Zhang Y. (2019). β2-adrenoceptor signaling reduction is involved in the inflammatory response of fibroblast-like synoviocytes from adjuvant-induced arthritic rats. Inflammopharmacology 27 (2), 271–279. 10.1007/s10787-018-0477-x 29675711

[B59] Xue X. Liu H. Wang S. Hu Y. Huang B. Li M. (2022). Neutrophil-erythrocyte hybrid membrane-coated hollow copper sulfide nanoparticles for targeted and photothermal/anti-inflammatory therapy of osteoarthritis. Compos. Part B Eng. 237, 109855. 10.1016/j.compositesb.2022.109855

