# Supplementary material for: Isoprenaline and salbutamol inhibit pyroptosis and promote mitochondrial biogenesis in arthritic chondrocytes by downregulating β-arrestin and GRK2
Source: Front Pharmacol. 2022 Sep 14;13:996321. doi: 10.3389/fphar.2022.996321 (PMC9519065; doi:10.3389/fphar.2022.996321)
Supplement: Supplementary file 1 [file Table1.docx]

**Supplementary Table 1**

**qPCR primer sequences:**

| Primer name |  | Sequence (5'-3') |
| --- | --- | --- |
| *In vitro* (Homo Sapiens) | | |
| *β2-AR* | F | GCCTGTGCTGATCTGGTCAT |
|  | R | AATGGAAGTCCAAAACTCGCA |
| *Bcl-2* | F | GGTGGGGTCATGTGTGTGG |
|  | R | CGGTTCAGGTACTCAGTCATCC |
| GLUT-1 | F | GGCCAAGAGTGTGCTAAAGAA |
|  | R | ACAGCGTTGATGCCAGACAG |
| *PGC-1α* | F | TCTGAGTCTGTATGGAGTGACAT |
|  | R | CCAAGTCGTTCACATCTAGTTCA |
| *NRF2* | F | TCCAGTCAGAAACCAGTGGAT |
|  | R | GAATGTCTGCGCCAAAAGCTG |
| *MMP1* | F | GGGGCTTTGATGTACCCTAGC |
|  | R | TGTCACACGCTTTTGGGGTTT |
| *MMP3* | F | CTGGACTCCGACACTCTGGA |
|  | R | CAGGAAAGGTTCTGAAGTGACC |
| *MMP9* | F | GGGACGCAGACATCGTCATC |
|  | R | TCGTCATCGTCGAAATGGGC |
| *ADAMTS5* | F | GAACATCGACCAACTCTACTCCG |
|  | R | CAATGCCCACCGAACCATCT |
| *COL2A1* | F | TGGACGCCATGAAGGTTTTCT |
|  | R | TGGGAGCCAGATTGTCATCTC |
| *Acan* | F | ACTCTGGGTTTTCGTGACTCT |
|  | R | ACACTCAGCGAGTTGTCATGG |
| *In vivo* (Mice ) | | |
| *PGC-1α* | F | AAGTGGTGTAGCGACCAATCG |
|  | R | AATGAGGGCAATCCGTCTTCA |
| *Bcl-2* | F | GAGAGCGTCAACAGGGAGATG |
|  | R | CCAGCCTCCGTTATCCTGGA |
| *MMP1* | F | CCTTGATGAGACGTGGACCAA |
|  | R | ATGTGGTGTTGTTGCACCTGT |
| *MMP3* | F | TCTGGGCTATACGAGGGCAC |
|  | R | ACCCTTGAGTCAACACCTGGA |
| *ADAMTS5* | F | CCCAGGATAAAACCAGGCAG |
|  | R | CGGCCAAGGGTTGTAAATGG |
| *NRF2* | F | CTTTAGTCAGCGACAGAAGGAC |
|  | R | AGGCATCTTGTTTGGGAATGTG |
| *COL2A1* | F | GGGTCACAGAGGTTACCCAG |
|  | R | ACCAGGGGAACCACTCTCAC |
| *β2-AR* | F | TGGGGCCAGTCACATCCTTAT |
|  | R | TGACGCACAACACATCAATGG |
| *Acan* | F | ATTTCCACACGCTACACCCTG |
|  | R | TGGATGGGGTATCTGACTGTC |
| *MMP9* | F | GGACCCGAAGCGGACATTG |
|  | R | CGTCGTCGAAATGGGCATCT |
| *β-arrestin* | F | AGTCGAGCCCTAACTGCAAG |
|  | R | ACGAACACTTTCCGGTCCTTC |
| *GRK-2* | F | AGCCCTTGGTGGAGTTCTAC |
|  | R | CCCCTCGGAGGTTCTGACA |

Sequence of primers used in this study.
