# Supplementary material for: Isoprenaline and salbutamol inhibit pyroptosis and promote mitochondrial biogenesis in arthritic chondrocytes by downregulating β-arrestin and GRK2
Source: Front Pharmacol. 2022 Sep 14;13:996321. doi: 10.3389/fphar.2022.996321 (PMC9519065; doi:10.3389/fphar.2022.996321)
Supplement: Supplementary file 2 [file Table2.DOCX]

**Supplementary Table 2**

**Comet assay:**

|  | **Sample** | **Comet length (µm)** | **Head**  **length (µm)** | **Tail**  **length (µm)** | **% DNA**  **in head** | **% DNA**  **in tail** | **Tail moment (µm)** |
| --- | --- | --- | --- | --- | --- | --- | --- |
| **A** | **Control** | 42.94±2.37 | 36.69±2.12 | 6.25±1.10 | 85.44±2.31 | 14.55±2.31 | 0.11±0.01 |
| **B** | **ISO** | 43.80±2.78 | 37.48±2.05 | 6.32±1.46 | 85.57±2.59 | 14.43±2.60 | 0.12±0.02 |
| **C** | **IL-1β** | 43.94±3.28 | 26.67±2.38 | 17.27±2.65 | 60.78±4.62 | 39.07±4.61 | 1.30±0.16 |
| **D** | **IL-1β+ISO** | 41.58±2.81 | 32.78±2.27 | 8.81±1.37 | 75.35±2.73 | 24.64±2.73 | 0.17±0.02 |

Nuclear fragmentation assessment of Human chondrocytes cell line (CHON-001) *via* comet parameters.
